# Optimizing the graft size in the Evans osteotomy to minimize the calcaneocuboid joint pressure by highly realistic in-silico analysis

**DOI:** 10.1038/s41598-025-85688-7

**Published:** 2025-01-21

**Authors:** Luca Quagliato, Youngbin Lim, Yunjeong Choi, Kyoung Min Lee, Sewon Kim, Taeyong Lee

**Affiliations:** 1 Division of Mechanical and Biomedical Engineering, Ewha Womans University, Seoul, 03760 Republic of Korea; 2 Division of Mechanical and Biomedical Engineering, Graduate Program in System Health Science and Engineering, Ewha Womans University, Seoul, 03760 Republic of Korea; 3 Dassault Systèmes Korea, SIMULIA, Seoul, 06164 Republic of Korea; 4 Dassault Systèmes Korea, CATIA, Seoul, 06164 Republic of Korea; 5 Department of Orthopedic Surgery, Seoul National University Bundang Hospital, Seongnam, 13620 Republic of Korea

**Keywords:** Medical research, Engineering, Mathematics and computing

## Abstract

This contribution details a new high-fidelity finite element analysis (FEA) methodology for the investigation of the effect of the graft size on the pressure distribution developing at the calcaneocuboid joint after the Evans osteotomy procedure. The FEA model includes all 28 bones of the foot up to the distal end of fibula and tibia as well as soft tissues, tendons, and muscles. The developed FEA model was validated by comparing the in-vivo pressure distribution on the foot plantar with the in-silico results, resulting in a low deviation equal to 7.8%, and with the deformed foot shape caused by the body weight in static standing position measured by a high-precision lidar scanning, for an average shape error of 5.3%. The developed FEA was then employed to investigate the effect of the graft size on the calcaneocuboid joint pressure and improvement in the medial-longitudinal arch for a flat foot patient, where the soft and hard tissues’ geometries were acquired by a CT scan. The results quantitatively demonstrated that the maximum pressure at the calcaneocuboid joint follows a quadratic trend where the minimum point also represents the best compromise to alleviate the flat foot condition while avoiding excessive pressure.

**Supplementary Information:**

The online version contains supplementary material available at 10.1038/s41598-025-85688-7.

## Introduction

The Evans osteotomy is a surgical procedure commonly used to correct calcaneus valgus deformity, also known as flatfoot^1,2^. While the procedure allows correcting the curvature of the medial longitudinal arch^2,3^ and the biomechanics of the midtarsal joint^4^, as well as enhancing quality of life^5^, post-operative outcomes are somewhat mixed. Although many patients experience positive improvement some report complications, including calcaneocuboid (CC) osteoarthritis, under- or over-correction, lateral foot pain, and even partial detachment of the graft from its bone socket^6^. Consequently, various studies were conducted over the years to investigate the post-operatory effects of Evans osteotomy, employing both ex-vivo and in-silico approaches.

Until the mid-‘80s, the consensus was that the Evans osteotomy was an effective way to restore functionality to the medial-longitudinal arch^4^ but only in the late ‘90s and early 2000s scholars begun to investigate its effect on the calcaneocuboid joint pressure^7^. These early investigations are predominantly and mostly exclusively based on ex-vivo experiments, and although were carried out by employing similar techniques, are conflictual in their conclusions. Some scholars have suggested that a non-optimal (oversized) graft may lead to joint arthrosis^7^, while others found no conclusive evidence to support the hypothesis that a lateral column lengthening (LCL) causes joint overload^8^. Same concerning ex-vivo studies, more recent discoveries shed light on LCL magnitude effect on the CC joint pressures and reported that an 8 mm graft results in a minimization of the joint pressures, which is turn minimizes the secondary risk of calcaneocuboid osteoarthritis while correcting the flat foot deformity^9^. In conjunction with this, the Evans osteotomy has also been associated with a tendency for the forefoot to supinate, with the relevant magnitude strongly associated with the size and shape of the graft employed^2,9,10^ as well as an overloading of the lateral foot, measured in terms of a variation of the plantar pressure^10^. In this regard, from the literature employing ex-vivo experiments, it is clear and well-documented that the size of the graft in the Evans osteotomy is crucial in determining the magnitude of the LCL as well as the reorientation of hard and soft tissue from hindfoot to forefoot.

Considering the complexity and limitations associated with ex-vivo studies, the early 2000s saw a transition from fully ex-vivo-based research to fully in-silico approaches or to a combination of both methodologies. To this end, the finite element analysis (FEA) has been employed to deepen the investigation and to acquire information of highly impractical locations, such as the measurement of the post-operation tension and deformation arising on soft tissues^11^. The state of the art of FEA modeling for the Evans osteotomy is represented by complex simulations including bones, cartilage, tendons, and soft tissues present in the human foot^12^ and up to the tibial and fibula distal ends^13^. However, to the best of the authors’ knowledge, these representations are based on the insertion of the graft in an already rotated foot model. In other words, the whole of hard and soft tissues is rotated beforehand to accommodate the graft and does not rotate because of the insertion of the graft itself. This steady state FEA representation of the Evans osteotomy results in a linear increase of the compressive stress at the interface between the graft and the cuboid bone, afterward transferred to the calcaneocuboid joint, which is not aligned with ex-vivo studies^9^, where the peak pressure follows a quadratic trend with a *minima* between the fleet foot (original) condition and an oversized graft. Besides, the graft insertion not only results in a lengthening of the lateral column^8^ but also in a realignment of the whole of hard and soft tissues and a consequent variation of the plantar pressure^10^ due to increased supination of the forefoot^9,10^.

Considering the extent and limitations of the available literature, the research hypothesis related to this contribution can be formulated in terms of the development of a high-fidelity FEA model, based on the computer tomography scan (CT) scan, capable of accurately simulating the ex-vivo findings and allowing for the identifications of the optimal LCL to correct the calcaneus valgus deformity while minimizing the CC joint pressure. Accordingly, this research presents a high-fidelity FEA modeling approach including all bones, cartilage, tendons, and soft tissues present in the human foot up to the ankle joint. The FEA model was validated by comparing the in-silico plantar pressure distribution with the experimental one and the FEA-predicted foot deformation in standing conditions with the 3-dimensional foot model acquired through high-resolution lidar scanning. Afterward, by extending the analysis to the CT scan of an adult female with flatfoot conditions, the FEA model was employed to determine the graft size allowing for the minimization of the CC joint pressure. The realignment of soft and hard tissues was actively modeled as a consequence of the Evans osteotomy graft insertion and is computed as a non-steady state transition between two steady states, namely the original flat foot condition and the post-surgery converged geometrical state until the elastic equilibrium for both hard and soft tissues is reached once again. The proposed approach includes all the features of the state-of-the-art FEA modeling^12,13^ and supersedes them in terms of adherence to reality thanks to the consideration of the non-steady-state transition considering the realignment of hard and soft tissues consequent to the Evans osteotomy graft insertion. In this regard, the developed FEA model simulates the osteotomy procedure and predicts the stress state after the procedure through hinge connector elements, not by simple widening of the lateral column. These improvements are essential to accurately simulate the kinematic realignment of the foot bones after the Evans osteotomy procedure as demonstrated by the good adherence of the FEA results with the literature ex-vivo peak pressure trend^9^.

## Results and discussion

**Fig. 1:**
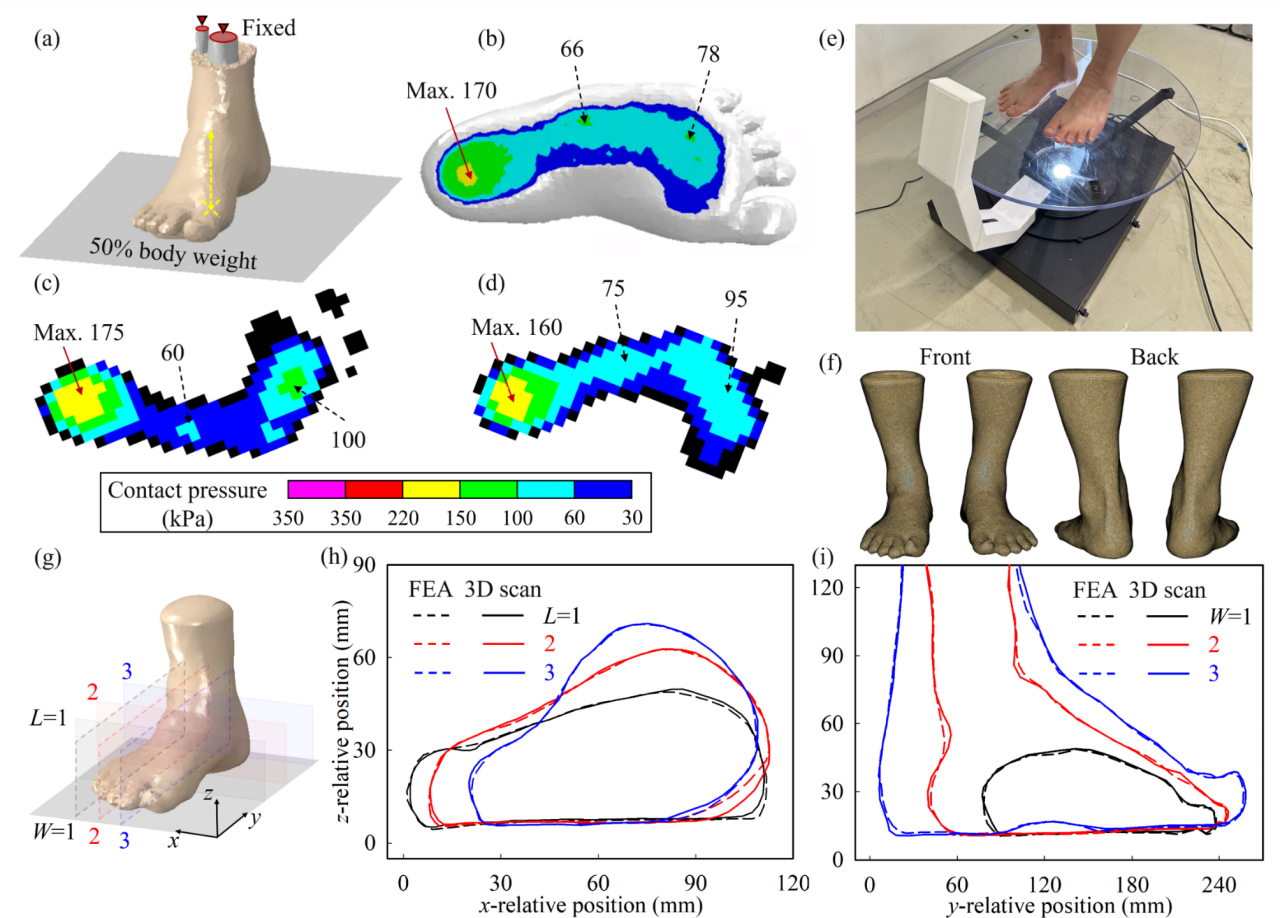
(**a**) Finite element analysis (FEA) model and schematic representation fixed and load conditions. Plantar pressure distribution from (**b**) healthy foot FEA simulation, Emed measurement for (**c**) right and (**d**) left foot. (**e**) MediACE3D lidar scanning of the same healthy foot subject and (**f**) exported 3D mesh file (front and back). (**g**) Criteria for the FEA and experimental foot profile analysis and comparison between 3D scan profile and FEA predictions along (**h**) length (*L*) and width (**i**) directions.

The FEA model of the healthy foot, relevant to an adult male in his 50’s with a foot size of 250 mm, has been employed as the blueprint for the simulation development, subsequently validated by comparing the foot shape, along median and coronal planes, and the pressure distribution developing at the foot plantar during balanced standing conditions. Half of the body weight was applied to the ground plate, while the distal end of the fibula and tibia, embedded in the whole soft tissues, were fixed, as shown in Fig. 1a. By assuming an equal redistribution of the body weight, only the right foot was included in the FEA model, Fig. 1b, whereas the pressure distribution was measured on both feet by the Emed (Novel GmbH) device. The comparison between the pressure distribution over the whole foot plantar shows good agreement between finite element and experimental results, Fig. 1c,d, with the maximum value located at the center of the hindfoot, estimated with a 6.3% deviation.

At this point, it is also interesting to highlight the fact that the experimental values highlight a difference between the right and left foot plantar pressure most likely linked to a difference in structure and soft tissues’ composition and standing pose balance, correctly modeled by the FEA analysis. In both experimental and FEA results for the right foot the midfoot experiences a fairly uniform pressure of 75 kPa, estimated by the FEA model at 66 kPa, with a deviation of 12%. Overall, although minor deviations are identified, the developed FEA modeling technique was demonstrated to be accurate in simulating the load transfer from the ankle to the foot plantar, thus the reliability of the material properties, simulation conditions, and reciprocal interactions between soft and hard tissues in the foot. The FEA model was further validated by comparing the geometry of the foot with the applied 50% of the body weight with the real 3D shape of the foot in standing conditions, measured with the MediACE3D (RealDimension, South Korea) high-resolution lidar foot scanner, Fig. 1e, where booth feet where scanned, Fig. 1f. By considering the right foot, three cross-sections along the length (*L*) and width (*W*) directions, Fig. 1g, located at 25%, 50%, and 75% of each direction, were considered in the validation and resulted in an average foot profiles deviations, equal to 6.3% and 4.7% respectively. The results are reported in Fig. 1h, i respectively. As a remark, it ought to be pointed out that, due to the in-vivo nature of this study, no intra-joint pressure could be measured. Nevertheless, considering the load path from its application surface to the fixed locations, Fig. 1a, the interactions between all the elements in between was indirectly validated through the two analyses above, proving the capability of the developed FEA model is accurately replicating the complex interaction of hard and soft tissue in the human foot.

**Fig. 2:**
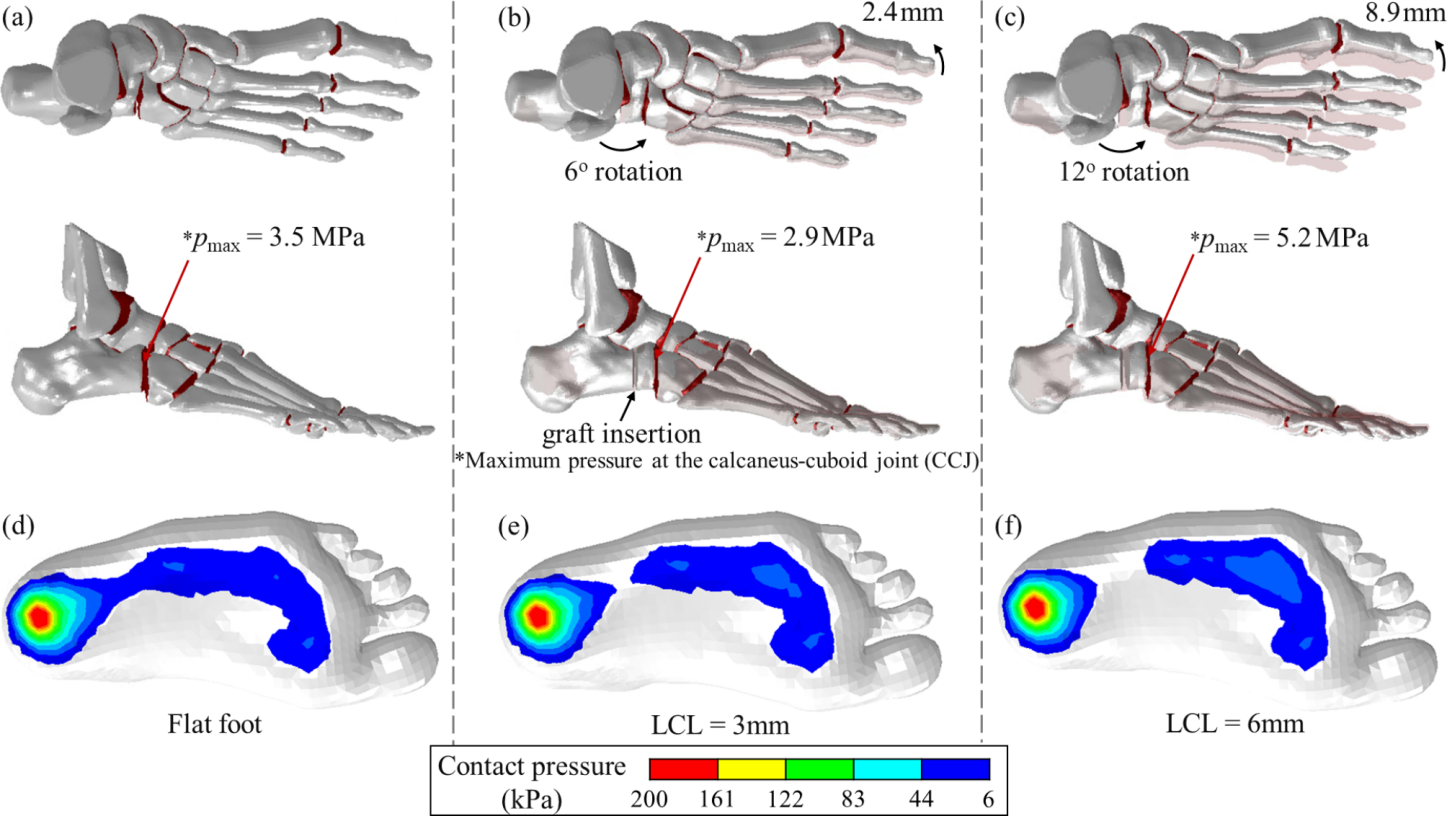
FEA results for the metatarsal reorientation and maximum pressure at the CC joint for (**a**) flat foot, (**b**) 3 mm LCL, and (**c**) 6 mm LCL from unloaded simulation. Plantar pressure distribution calculated from the loaded Case-2 simulation model for (**d**) flat foot, (**e**) 3 mm LCL, and (**f**) 6 mm LCL showing the rising of the midfoot arch as a consequence of the Evans osteotomy.

The developed and validated FEA model was then applied to a 235 mm size female individual affected by flat foot conditions with aim to investigate the influence of the graft size, also referred to as LCL, on the kinematic of the bones, as reported in Fig. 2. As shown in previous studies^2,3^, the Evans osteotomy procedure directly affects the lateral column, which results in lengthening to correct the flatfoot (pes planus) deformity but also in a rotation of the metatarsals, as shown in Fig. 2b,c.

This slight rotation is crucial in promoting a better redistribution of the pressure distribution across the foot during gait, helps in

improving the functionality of the foot, and reduces the symptoms associated with the flatfoot deformity. In this regard, the comparison between the original, Fig. 1a, and post graft insertion conditions, Fig. 2b,c, shows that doubling the graft size results in a metatarsal rotation 3.7 times higher, suggesting a more than a linear correlation with the graft size. Such huge rotation of the forefoot might also lead to overcorrection, thus in the arising of metatarsalgia, altered gait patterns, or stress on adjacent joints. In addition to that, as shown in Fig. 2, the insertion of a 3 mm graft results in a ~ 18% reduction of the maximum pressure in the CC joint, associated with an improvement in the plantar pressure, Fig. 2e. However, doubling the graft size generates a 1.5 times increase in the peak pressure, in comparison to the original flat foot condition as well as an overcorrection of the arch can be hypothesized from the plantar pressure distribution of Fig. 2f. Overall, the analysis of the results of Fig. 2 shows that part of the rotation caused by the graft insertion results in the improvement of the medial longitudinal arch curvature^2,3^ caused by a rotation of the whole metatarsal region of the foot. However, it also affects the intra-joint pressure, a fact that might affect cartilage wear, gait comfort, and overall food mobility and flexibility.

**Fig. 3:**
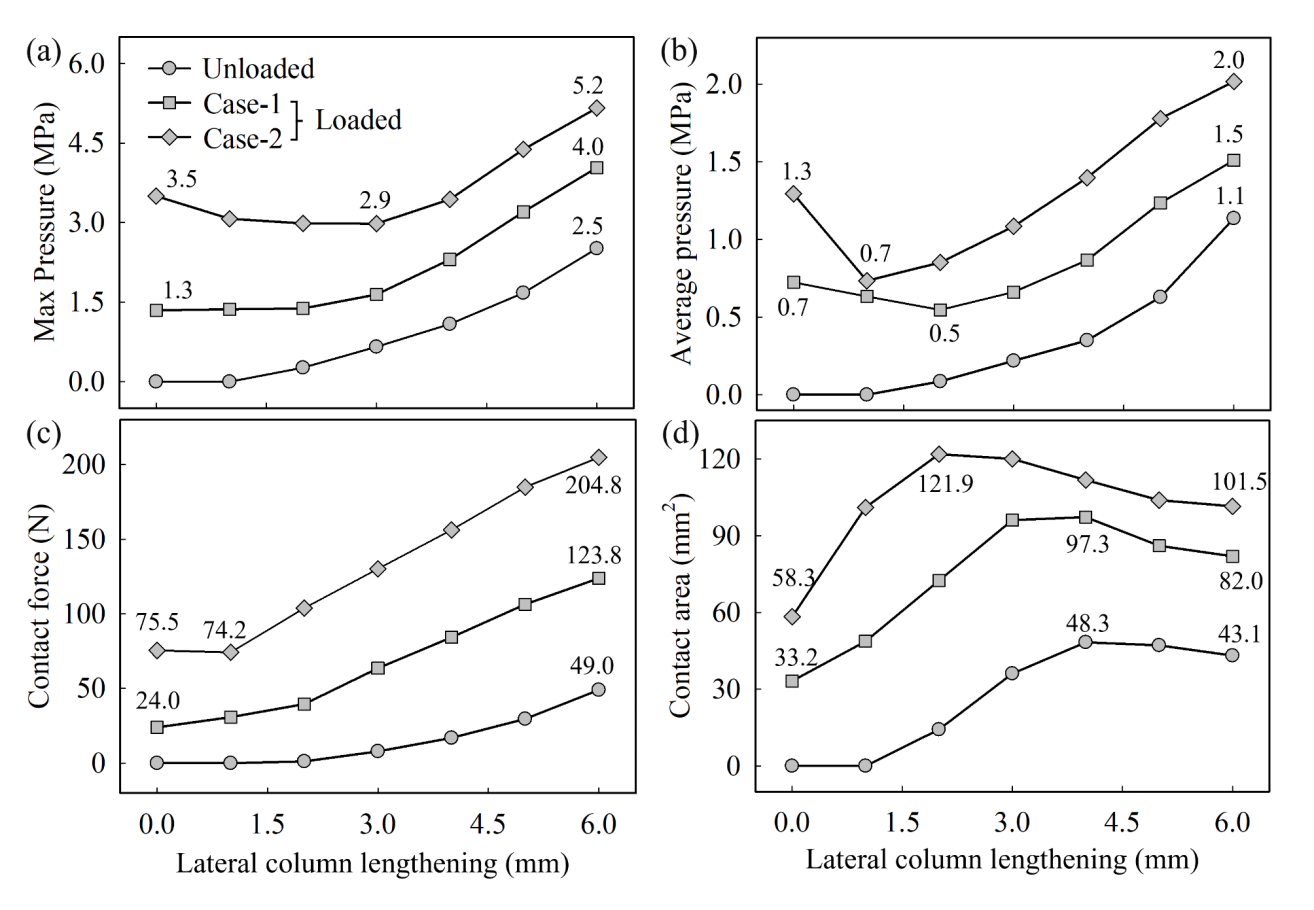
Effect of graft size on calcaneus-cuboid joint (**a**) Maximum pressure, (**b**) Average pressure, (**c**) Contact force, and (**d**) Contact area. The legend Unloaded represents FE prediction for Evans osteotomy, but not loaded with body weight. Case-1 (Loaded) and Case-2 (Loaded) represent FE prediction without & with tendon force, respectively.

**Fig. 4:**
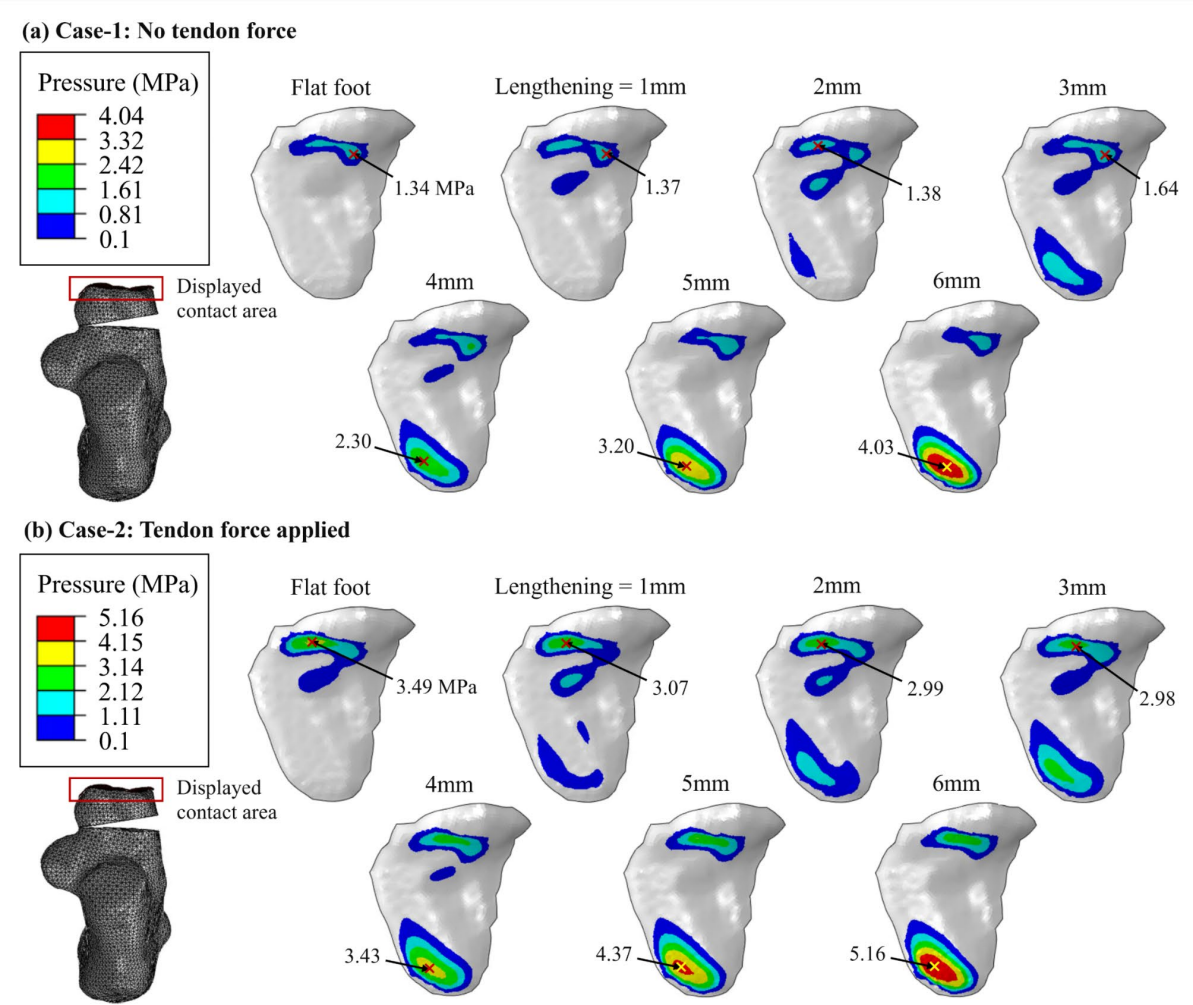
Variation of the contact pressure distribution in CCJ with the graft size (LCL) and considering the (**a**) case without muscle force, Case-1 (Loaded), and (**b**) where the muscle force is applied to the bones through the tendons, Case-2 (Loaded).

Analyzing further the effect of the LCL graft, but focusing on the calcaneocuboid joint, an important remark concerning the modeling approach ought to be brought forward. In the development of the FEA model two approaches have been employed: the former (Case-1), aimed at simplification, did not include the muscle activation forces, whereas the latter (Case-2) fully replicates the structure of the human foot and includes the muscles forces, modeled in terms of tendon force transferred from the muscles to the bones. In addition, the unloaded foot condition was considered the benchmark for the pressure distribution developing at the CC joint when the foot is fully unloaded. In the analysis, the effect of employing six graft sizes equal to 1, 2, 3, 4, 5, and 6 mm on the maximum and average pressures and the contact force and area in the CC joint was analyzed. From the results, reported in Fig. 3, various information can be elicited. First, for both unloaded and loaded cases, the contact force is minimal in the flat foot condition, Fig. 3c, meaning that the insertion of the graft inevitably results in an increase of the compressive force acting on the CC joint. However, when considered together with the contact area, Fig. 3d, it results in local *minima* for the maximum and average pressures, as reported in Fig. 3a,b. This fact can be explained by the aforementioned combined effect of the internal joint-cartilage deformation and metatarsal reorientation that, in the investigated patient’s case, find their optimal balance for a graft size of 3 mm. Moreover, the difference in modeling between the unloaded and two loaded (Case-1, Case-2) cases are clear and reflects the load transfer caused by the body weight throughout the foot joints, including the CC joint. Nonetheless, as shown in the comparison between “Case-1 (Loaded)” and “Case-2 (Loaded)”, the addition of the muscle force in the foot modeling, transferred in terms of tendon load to the bones, allows for a peculiar and yet preferable behavior for the maximum pressure. *Id est*, in the 2 to 4 mm graft size range, the maximum pressure experiences a local *minima*, which is not predicted for the other two simulation conditions but measured in ex-vivo studies^9^. Although the muscle force is considered a constant value, and applied as tendon forces, its effect on the contact conditions in the CC joint is the redistribution of the compression force over a wider area in the region where the max pressure is likely to arise, as summarized in Fig. 4.

As the graft size increases, the cuboid is pushed forward increasing the pressure on the CC joint, especially on the outer side of the foot. In this scenario, most of the deformation, and resulting pressure, arises on the CC joint whereas the highest rotation is experienced by the big toe, Fig. 2. In this regard, the increase in the graft size results in a redistribution of the pressure from a single area of contact, as in the flat foot condition, towards the side and ultimately to the bottom of the joint area, Fig. 4. Moreover, either not considering or including the muscle action by means of tendon forces, as reported in Fig. 4a, b respectively, results in a progressive reduction of the ratio between the maximum pressure between the latter and the former. In other words, for small graft sizes, the effect of the muscle forces transferred to the foot bones creates a sort of obstacle for the reorientation of the metatarsals, thus in a higher peak pressure in the CC joint. However, for greater graft sizes, the reaction force exerted by the tendons is overcome by the magnitude of the LCL, a fact that might result in gait change and post-operatory issues, especially for the plantar fascia. In addition to that, for the results of both Fig. 4a,b, it can be grasped that the flat foot condition makes the pressure distribution in the CC joint be located mostly on the top of the joint, whereas for increasing graft sizes, the peak pressure shifts to the bottom of the contact area. It ought to be pointed out that, although not directly displayed in this research, the quadratic-like correlation between the graft size and the maximum pressure in the CC joint is influenced by the soft tissue surrounding the joint, considered in this research and proved in the literature ex-vivo studies^9^.

Recent contributions dealing with FEA modeling of the Evans osteotomy did not calculate the post-insertion equilibrium and did not include either the muscle/tendon activation force^12^ or model the soft tissues surrounding the foot^13^. Although similar pressure contours like the one in Fig. 4 were obtained, the correlation with the graft size was determined as linear, thus not in agreement with experimental observations^9^. As demonstrated in this research, the identification of the correct LCL, in terms of the global minima for the maximum pressure in the CC joint, requires a narrow interval between graft size conditions, which was set to 1.0 mm in this study, whereas 2.5 mm and 2 mm in the recent literature^12,13^. In this regard, and according to the high-fidelity of the FEA model proposed in this research, the identification of the correct graft size for the Evans osteotomy can be treated as an FEA tradeoff problem where the optimal size is represented by a compromise between minimizing the CC joint pressure, as in Fig. 4b, while allowing for the creation of a sufficient midfoot arch, Fig. 2e. For the flatfoot patient considered in this research, this was achieved by an LCL = 3 mm, but indeed an adaptation of the proposed approach must be carried out to apply it to each specific patient. Accordingly, the proposed approach should serve as a blueprint for implementing a patient-specific FEA model based on the relevant CT scan, although the adaptation would require some effort for the setting of a new FEA simulation. It ought to be pointed out that the utilization of reference values for the material properties of hard and soft tissues was made necessary due to the in-vivo nature of this study, and although it resulted in good accuracy, additional characterization with more modern equipment would improve it even further.

From a clinical perspective, although calcaneal lengthening is an effective procedure for correcting the three-dimensional deformity of flatfoot, it has been criticized for its potential to increase calcaneocuboid joint pressure. This study suggests that the calcaneocuboid joint pressure does not inevitably increase with calcaneal lengthening and shows that there is an optimal amount of lengthening that minimizes calcaneocuboid joint pressure. Applying this to the surgical setting, when performing corrective surgery for flatfoot deformity, implies that calcaneal lengthening should be performed up to the point where calcaneocuboid joint pressure is minimized. If any residual deformity remains, additional procedures such as medial displacement calcaneal osteotomy or Cotton osteotomy (dorsal opening wedge osteotomy of the medial cuneiform) may be performed to achieve the desired correction and reduce complications associated with increased calcaneocuboid joint pressure. In this regard, the proposed approach can be extended to other patients to help orthopedic surgeons with pre-operative planning to tailor the graft size and positioning to each patient’s unique anatomy, although an interdisciplinary effort is indeed required. It is also worth highlighting that clinicians require clear, validated outcomes from any new technology, meaning that before the proposed approach can be safely included in pre-surgery planning, additional in-vivo validations ought to be carried out to gather more data for a pre-and post-surgery statistical analysis allowing for a more comprehensive understanding of both strong points and limitations of the proposed approach.

Nevertheless, this study demonstrated the feasibility of employing the developed FEA model for a patient-specific identification of the best graft size in the Evans osteotomy to allow for the improvement of the plantar pressure, through the creation of the medial arch, while limiting the CC joint pressure distribution. In conclusion, the developed FEA model represents an important step forward and bolsters the ever-growing interdisciplinarity between engineering and medicine. Indeed, ex-vivo characterizations are crucial to set the benchmark, but only in-silico modeling, as the FEA employed in this research, possesses the generalization capability to be deployed in a patient-specific scenario. For these reasons, the methodology and results presented in the research might be of interest to clinicians, podiatrists, and biomechanical engineers involved with foot research and anamnesis.

### Methods

#### Finite element modeling

In this study, the commercial finite element solver Abaqus/Standard (2023 version) was adopted for the simulation of the balanced standing and the Evans osteotomy procedure. This section outlines the construction of finite element mesh, material modeling, boundary conditions applied in the model and the solver setting.

## Finite element mesh

**Fig. 5:**
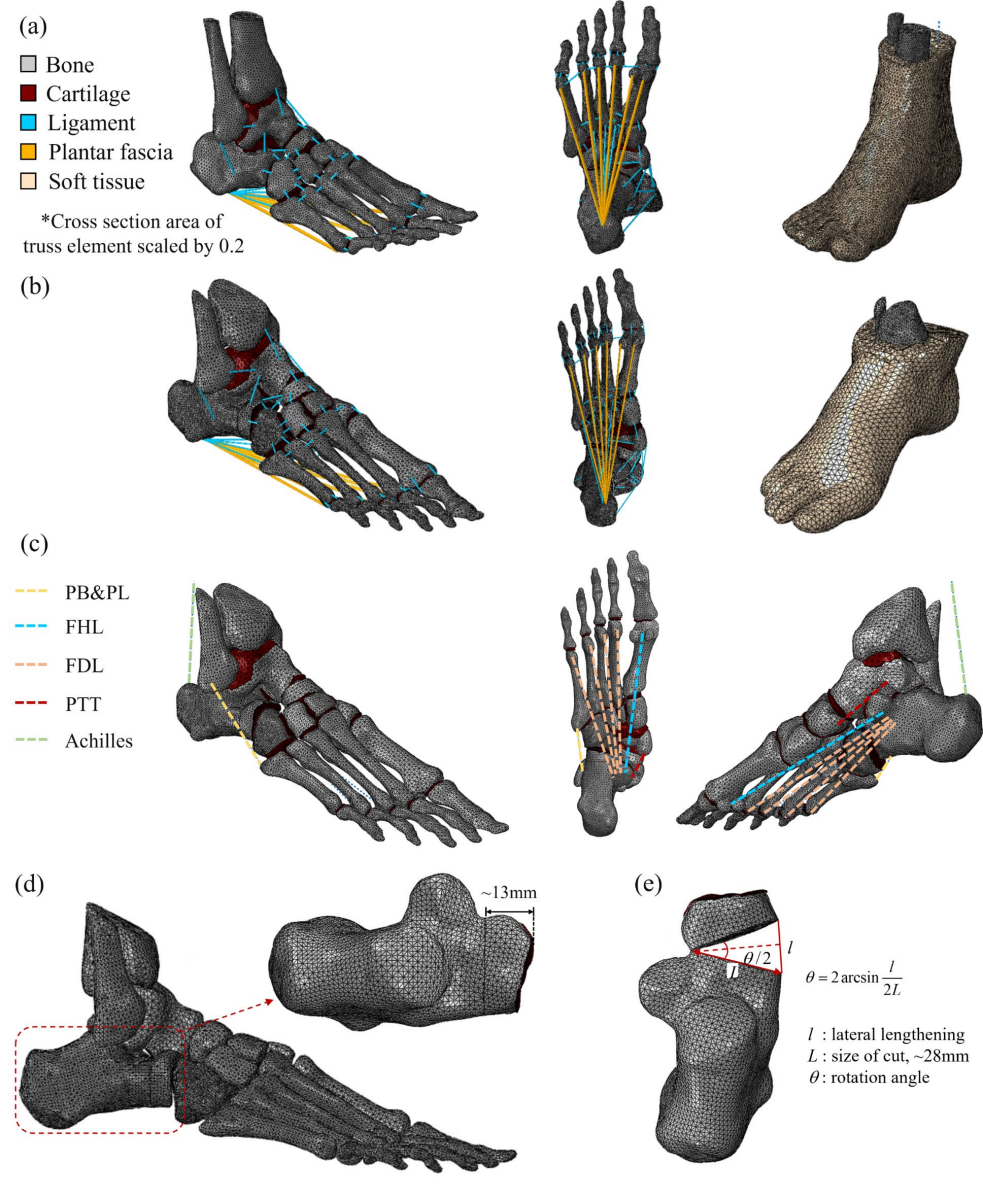
FEA models summary. (**a**) Non-flat foot, (**b**) Flat foot, (**c**) Tendons modeled as connector elements in the flat foot FEA model. Ligaments and fascia hidden for visualization. The PB&PL represents peroneus brevis and peroneus longus respectively. The FHL, flexor hallucis longus, FDL, flexor digitorum longus, PTT is posterior tibial tendons (**d**) Location of Evans osteotomy site and (**e**) relation between rotation angle and lateral column lengthening.

Figure 5 shows finite element mesh of the foot models, a non-flat foot model for the validation of FEA model, Fig. 5a, and a flat foot model for the simulation of Evans osteotomy procedure, Fig. 5b, where 690k and 550k elements were used respectively. A total of 28 bones up to the tibia and fibula distal ends were meshed with C3D4 tetrahedral elements from the CT-scanned geometry with the average element size of 2.5 mm. The soft tissue was also meshed with C3D4 elements but with a smaller average mesh size of 2.0 mm. The cartilage was meshed with an average of 2.5 mm and constructed by stacking five C3D6H wedge elements in the thickness direction, perpendicular to the bone surface. The hybrid element formulation was chosen to accurately capture the nearly incompressible nature of cartilage material. Ligament and fascia were modeled with 1D truss element^14^, an hypothesis validated by comparing plantar pressure distribution from the full 3D model. Thus, a total of 74 ligaments and 6 fasciae were modeled with T3D2 truss elements. The connecting points of each T3D2 truss elements were determined from the CT-scanned foot anatomy and FE model of the previous studies^14–16^.

## Material modeling

Since in-vivo measurement for the material property of human foot is challenging, material parameters were adopted from the previous studies and summarized in Table 1 whereas the ground plate was modeled as discrete rigid. The bone and cartilage were modeled as isotropic and linear elastic material according to previous studies^17,18^. For the ligament, and plantar fascia, isotropic elastic material model was chosen and assumed to be exclusively tensile-resistant. It can be achieved in Abaqus/Standard in combination of *No compression option for the *Elastic keyword and the use of truss element which has no bending stiffness.

**Table 1:** Material model and parameter for the foot model.

Part	Material model	Material property	References
Bone	Linear elastic	*E* = 7.3 GPa, *v* = 0.3	Chen et al.^17^
Cartilage	Linear elastic	1 MPa, *v* = 0.4	Athanasiou^18^
Ligament	Linear elastic Compression only	*E* = 260 MPa, *v* = 0.4, Cross section area = 18.4 mm^2^	Siegler et al.^19^
Plantar fascia		*E* = 350 MPa, *v* = 0.4, Cross section area = 58.6 mm^2^	Kitaoka et al. ^20^
Soft tissue	Hyperelastic Yeoh model	*C*_10_ : 2.26E-2 MPa, *C*_20_ : -8.61E-3 MPa, *C*_30_ : 6.25E-3 MPa, *D*_1_ : 3.65 MPa^− 1^, *D*_2_ : 46.4E4 MPa^− 1^, *D*_3_: 5.0E3 MPa^− 1^	Calibrated from Lemmon et al. ^21^

The material parameter and cross section area of ligament and fascia were obtained from previous studies^19,20^, respectively. Regarding the soft tissue, the hyperelastic material parameter calibrated from in-vivo uniaxial compression test by Lemmon^21^ is widely used in foot FEA models^14,15^, which is second order polynomial strain energy potential with parameter *C*_10_: 8.55E-2 MPa, *C*_01_: -5.84E-2 MPa, *C*_11_: -2.32E-2 MPa, *C*_20_: 3.9E-2 MPa, *C*_02_: 8.51E-3 MPa, *D*_1_: 3.65E-3 MPa^-1^. However, the literature material properties for the soft tissue^21^ present an instability at uniaxial strain larger than 0.27 and smaller than − 0.84, which limits its range of application. If the strain in the soft tissue reaches these values, Abaqus/Standard solver has difficulties in converging. For the material model to be numerically stable, the Drucker stability condition must be satisfied as in Eq. (1).

1 $$d\sigma :d\varepsilon > 0$$

Here, *d****σ***,* d****ε*** is the infinitesimal change in the true stress and strain respectively. Using the relation *d****σ *** = **D** : *d****ε***, where **D** is the material tangent stiffness matrix, the inequality becomes:

2 $$d\varepsilon :{\mathbf{D}}:d\varepsilon > 0$$

From Eq. (2), it is required that the matrix D to be positive definite. Since the soft tissue is nearly incompressible, any hydrostatic stress can be chosen for the calculation of Eq. (2). If we choose *σ*_3_ = *dσ*_3_ = 0, Eq. (2) becomes Eq. (3), where *D*_ij_ is a function of stretch ratio *λ*_1_,* λ*_2_,* λ*_3_. For the positive-definite matrix:

3 $$\left[ {\begin{array}{*{20}c} {d\varepsilon _{1} } & {d\varepsilon _{2} } \\ \end{array} } \right]\left[ {\begin{array}{*{20}c} {D_{{11}} } & {D_{{12}} } \\ {D_{{21}} } & {D_{{22}} } \\ \end{array} } \right]\left[ {\begin{array}{*{20}c} {d\varepsilon _{1} } \\ {d\varepsilon _{2} } \\ \end{array} } \right] > 0$$

For the positive-definite matrix, *D*_11_ *+ D*_22_ *> 0* and *D*_11_*D*_22_ − *D*_12_*D*_21_ > 0, checked for the three deformation modes: uniaxial, planar, biaxial loading when Abaqus/CAE material evaluate feature^22^ has been employed. In this study, the Yeoh model, 3rd order reduced polynomial strain energy potential was chosen to satisfy the Drucker stability condition for all strain range. The material parameter was re-calibrated from uniaxial compression test data and the volumetric stress-strain curve predicted with the original hyperelastic model and compared with the literature results^21^ provided as supplementary material.

## Load and boundary conditions

The general contact formulation in Abaqus/Standard solver was adopted for the contact simulation. With this contact formulation, surface pairs are automatically detected and engaged in the contact without defining all possible contact pairs^21^. The frictional contact was used for the interaction between ground plate and soft tissue, and coulomb friction model with friction coefficient, *µ* = 0.6 was applied^13,14^. The bone and soft tissue are assumed to be fully bonded based on previous studies^13,14^, meaning that no relative motion between bonded surface can occur. The contact with cartilage surface is normally considered as well-lubricated. Thus, frictional contact with very small friction coefficient, *µ* = 0.001 was assumed^13,14^. Finally, interaction between soft tissue and ligament, fascia was simplified by excluding truss element surface in general contact domain.

To simulate the tendon force during the balanced standing, Achilles, peroneus brevis and peroneus longus (PB&PL), flexor digitorum longus (FDL), flexor hallucis longus (FHL), and posterior tibial tendons (PTT) were modeled as axial connector elements as shown in Fig. 5c. The compressive load in axial direction of the two connecting points can be applied through the axial connector. The connector loads for each tendon are summarized in Table 2 and are based on previous studies^9,22^.

It must be noted that PTT is not loaded in flat foot case due to PTT dysfunction^9^. On the other hand, the FDL is not loaded, and PTT is loaded instead for non-flat foot and lateral column lengthening (LCL) case after Evans osteotomy^9^. The Achilles tendon is loaded with 25% of bodyweight load^23^. In this study, the connector elements are assumed to have no mass and stiffness for simplicity. Regarding the boundary conditions, upper surface of tibia, fibula and one end point of the Achilles connector was fixed in all degree of freedom (DOF) as shown in Fig. 1a. The DOF of the ground plate was coupled with the reference point located in the plate center where DOF in surface normal direction is available only. Half of the body weight load was applied in the reference point of the ground plate.

**Table 2:** Connector load applied for the simulation of tendon force during balanced standing.

Tendon	Load (*N*)	Flat foot	Non-flat foot, LCL	Reference
Achilles	− 125	O	O	Lewis^23^, 25% of body weight
PB&PL	− 35	O	O	Xia et al. ^9^
FHL	− 22	O	O	–
FDL	− 5.5 × 4	O	X	Healed FDL transfer^9^
PTT	− 40	X	O	PTT dysfunction^9^

### Evans osteotomy simulation

Considering the far superior elastic modulus of the graft compared to the surrounding soft and hard tissues (elastic modulus of steel: 200GPa, bone: ~10GPa, soft tissue: ~100 kPa respectively), the inserted wedge-shaped graft can be considered as rigid body motion of the two osteotomy surfaces. Thus, the graft insertion for the Evans osteotomy procedure was simplified by applying relative rotational motion between osteotomy surfaces which are located 13 mm away from the calcaneus-cuboid (CC) joint as shown in Fig. 5d. By considering the graft insertion as a boundary condition between the two surfaces, instead of the physical insertion of an addition element in the FEA simulation, allows for avoiding complex contact computation between elements, which would inevitably introduce the issue of defining the friction conditions between the tissues and the graft during the transients of the insertion process.

Accordingly, the relative rotation between two surfaces was modeled using hinge connectors element in Abaqus having only one DoF, i.e. the x-axis rotation and the remaining 5 DoFs are all constrained^21^. As connector elements can only define relative motion between the two points, each osteotomy surface was coupled to a reference point located at the hinge axis using a kinematic coupling constraint, meaning that the surfaces behave like rigid bodies. To apply rotational boundary conditions for the hinge connector in Abaqus, the LCL needs to be converted to a rotation angle. The LCL, *l*, has following relation with rotation angle *θ* of Fig. 5e where *L* is the size of cut, equal to 28 mm.

4 $$l = 2L\sin \frac{\theta }{2}$$

In this study, the LCL range of 0 ~ 6 mm, corresponding to 0º ~ 12.3º, was considered for the Evans osteotomy procedure, where 0 mm LCL represents the flat foot condition. The natural equilibrium state of flat foot after Evans osteotomy can be predicted by applying relative motion, instead of absolute rotation boundary condition from global coordinate system.

## Solver setting and FEA convergence criteria

Since the Evans osteotomy was simulated with rigid body rotation of the two osteotomy surfaces, *Static, general solver in Abaqus/Standard may have difficulties in obtaining the converged solution. Thus, the dynamic, implicit solver with backward Euler time integration was chosen for the better convergence. It can be achieved by using *Dynamic, application = quasi-static keyword in Abaqus. In quasi-static dynamic solver, the inertia in the model have numerical damping effect making it easier for the solver to determine the converged solution for the problems involving rigid body motion or sudden decrease in the structural stiffness^21^. The total time period of the Evans osteotomy procedure and the balanced standing was set to 1 s each. However, since the inertial effect in quasi-static dynamic solver is numerical rather than physical, the 1 s of the step time should be interpreted as numerical value. The minimum and the maximum time increment was set to 1E-9, and 0.1 s, respectively. Regarding the convergence criteria, the default setting provided in Abaqus was used where the convergence is to be achieved for each time increment when the residual out-of-balance force is smaller than 0.5% of the time averaged force^21^, with the maximum number of attempt for each time increment was increased from 5 to 10 as the model involves many contact.

## Foot plantar pressure distribution experiments

To verify the accuracy of the implemented FEA model for the non-flat foot, static pressure test employing the Emed XL device, have been carried out. The employed pressure mattress is composed of 25,344 individual sensors capable of detecting the local pressure distribution throughout the whole foot plantar, subsequently post-processed by the Emed Expert software. The experiments have been carried out considering the same male subject in his 50’s whose foot model was employed for the definition of the FEA model presented in the *material modeling* section. The results, in terms of foot plantar distribution, are reported in Fig. 1c,d, employed for the validation of the FEA model in the results section.

## Electronic supplementary material

Below is the link to the electronic supplementary material.

Supplementary Material 1

## Data Availability

Raw data, the finite element model developed in the ABAQUS environment, and the results presented in this paper are all made available on request to the corresponding author.
