# Supplementary material for: Optimizing the graft size in the Evans osteotomy to minimize the calcaneocuboid joint pressure by highly realistic in-silico analysis
Source: Sci Rep. 2025 Jan 21;15:2668. doi: 10.1038/s41598-025-85688-7 (PMC11751300; doi:10.1038/s41598-025-85688-7)
Supplement: Supplementary file 1 — Supplementary Material 1 [file 41598_2025_85688_MOESM1_ESM.pdf]

Supplementary material

The material calibration result for the soft tissue material outlined in the method – Finite element modeling – material modeling section is shown here. In this study, Yeoh model, 3rd order reduced polynomial strain energy potential was chosen to satisfy Drucker stability condition for all strain range. The material parameter was re-calibrated from uniaxial compression test data by Lemmon *et al*[21], and volumetric stress-strain curve predicted with the original hyperelastic model proposed by Lemmon *et al*. The stress-strain curves are compared in figure S1. The stress-strain curves show good agreement for both deformation modes. The calibrated material parameter is mentioned in the material modeling section of the manuscript.

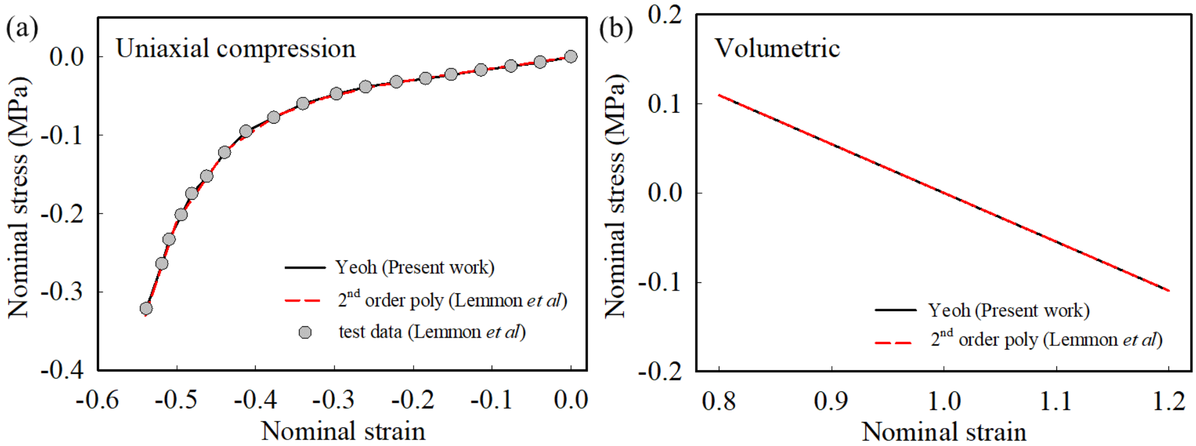

Figure S1. Material calibration results. (a) Uniaxial compression (b) Volumetric deformation.
